# Fault Detection and Monitoring in Induction Machines Using Data-Driven Model Drift Detection

**DOI:** 10.3390/s26051595

**Published:** 2026-03-04

**Authors:** Abdiel Ricaldi-Morales, Camilo Ramírez, Jorge F. Silva, Manuel A. Duarte-Mermoud, Marcos E. Orchard

**Affiliations:** 1 Department of Electrical Engineering, University of Chile, Av. Tupper 2007, Santiago 8370451, Chile; camilo.ramirez@ug.uchile.cl (C.R.); josilva@ing.uchile.cl (J.F.S.); morchard@ing.uchile.cl (M.E.O.); 2 Facultad de Ingeniería y Arquitectura, Universidad Central de Chile, Av. Santa Isabel 1186, Santiago 8330601, Chile

**Keywords:** induction motors, stator short-circuit faults, mutual information, model drift detection, fault detection

## Abstract

Stator short-circuit faults (SSCFs) account for a significant portion of induction motor failures, yet their early detection remains a challenge in industrial environments where labeled fault data is scarce and installing additional sensors is often impractical. This paper proposes a novel, data-driven fault detection and diagnosis framework grounded in the Residual Information Value (RIV) principle to overcome reliability limitations of traditional spectral and residual energy methods. By redefining fault detection as a statistical test of independence between control inputs (voltages) and current residuals, the proposed method identifies incipient faults as model drifts without relying on prior knowledge of fault distributions. A key contribution of this work is the seamless integration of the diagnostic scheme into standard Variable Speed Drives (VSDs): the healthy nominal model (a Multilayer Perceptron) is trained exclusively using data from the drive’s existing self-commissioning routine, eliminating the need for manual data collection or complex physical parameter identification. Experimental validation on an industrial test bench demonstrates that the framework achieves superior diagnostic performance compared to traditional baselines, providing higher statistical separability and a reduced false alarm rate. The system can detect 1% incipient faults in approximately 61 ms while accurately identifying the faulty phase. The results confirm that the proposed RIV-based strategy offers a robust, non-intrusive, and industry-ready solution for predictive maintenance that effectively balances high-speed detection with enhanced statistical reliability.

## 1. Introduction

Induction motors are among the most critical assets in modern industry due to their robustness, relatively low cost, and simple construction, which have led to their widespread adoption across a wide range of applications [1,2,3]. Given their central role in industrial processes, the reliability of these machines is of paramount importance. Faults in induction motors not only reduce efficiency but can also trigger unplanned shutdowns, with severe economic consequences in the case of catastrophic failures [4,5]. For this reason, predictive maintenance strategies that enable the early detection of incipient faults are essential, as they allow corrective actions before faults evolve into severe or irreversible damage. Therefore, predictive maintenance strategies are essential, as they enable early detection and timely corrective actions before faults evolve into severe or irreversible damage.

Induction motor failures are typically classified into stator, rotor, bearing, and miscellaneous faults, with stator faults (SFs) drawing particular attention due to their rising incidence, representing more than 30% of induction motor failures [5,6,7]. Such statistics highlight the importance of developing reliable diagnostic techniques for stator-related faults, especially under realistic operating conditions where voltage imbalance, load variability, and non-Gaussian disturbances are frequently present. In this context, the capability to detect faults across diverse operating regimes—including light loading or variable speeds—is essential. Such faults are technically defined as “incipient” when they involve a limited number of shorted turns (typically less than 3% of the phase winding) [4]. Unlike severe failures, incipient faults do not immediately trigger conventional protection systems; instead, they generate localized hotspots that progressively degrade the insulation, inevitably leading to catastrophic breakdown if left unaddressed [1].

Recent research on induction motor fault detection (FD) has primarily advanced along two methodological directions, data-driven and model-based approaches, with hybrid techniques also emerging in recent years. Data-driven methods rely exclusively on signal measurements and employ statistical or machine learning tools to infer the health state of the machine, without requiring a detailed physical model of its dynamics. Conversely, model-based approaches exploit mathematical representations of the motor—such as equivalent circuits or state-space models—to identify discrepancies between expected and observed behavior. Finally, hybrid approaches combine signal-driven indicators with theoretical models to enhance robustness and interpretability.

In the realm of data-driven strategies, machine learning has played a transformative role in fault detection across many fields, including induction motor diagnosis [4,5,6,7,8,9,10,11,12,13]. Surveys such as [4,5] provide comprehensive overviews of these techniques, most of which rely on artificial neural networks (ANNs), recurrent neural networks (RNNs), gated recurrent units (GRUs), long short-term memory (LSTM) networks, and convolutional neural networks (CNNs) and their variants. In particular, ref. [4] highlights diagnostic methods that monitor mainly current signals (and occasionally voltage) under both line-fed induction motors and variable-speed drives (VSD), covering stator faults and other failures. Meanwhile, ref. [5] specifically addresses induction motors controlled by direct torque control (DTC), emphasizing the challenges posed by stator-related faults and their effect on system reliability.Crucially, it highlights that the closed-loop control action tends to compensate for the fault effects, masking the symptoms and making detection significantly more challenging than in open-loop systems.

Several works have proposed machine learning schemes tailored for different induction motor operating conditions. For instance, in the context of direct field-oriented control (DFOC) drives, ref. [8] introduced an integrated method combining hierarchical CNNs with support vector machines (SVMs), optimized by particle swarm optimization (PSO), where current signals are transformed into images for incipient fault detection. Similarly, for induction motors under open-loop *V*/*f* control, ref. [6] developed a CNN-based FD algorithm complemented by encoder measurements. Addressing grid-connected induction motors, ref. [9] explored two composite architectures—CNN-LSTM and CNN-GRU—under balanced and unbalanced supply conditions.

A prominent trend involves image-based transformations of current signals. For example, ref. [11] employed CNNs fed with images from phasor magnitudes of fundamental components. Along the same line, ref. [12] proposed a recurrence plot CNN (RPCNNet) that encodes three-phase currents into RGB recurrence plots after Park transformation. In a more advanced control setting, ref. [7] analyzed an induction motor controlled by model predictive control (MPC) using RNN and CNN classifiers. On a different complexity scale, ref. [13] demonstrated a simpler yet effective strategy using a k-NN classifier on PCA-reduced signals, implemented in real-time on an FPGA.

Regarding hybrid approaches—which integrate the aforementioned signal-driven indicators or theoretical models with machine learning—distinct strategies have been proposed to enhance diagnostic performance [1,2,3,14,15,16,17]. A common strategy in this domain is a two-stage process of feature extraction followed by classification.

For instance, refs. [1,2] adopt this two-stage strategy using a mutual information estimator between delayed stator currents as a feature, classified with decision trees (DTs) or multilayer perceptrons (MLPs). However, these works do not establish a rigorous theoretical connection between mutual information and the physical fault mechanism. Both approaches were tested in line-fed induction motors, with [2] also demonstrating feasibility in industrial environments using a TMS320F28388D microcontroller. Similarly, ref. [3] presented a real-time method for a VSD-fed induction motor using Discrete Wavelet Transform (DWT) and the L2 norm of statistical parameters for feature extraction, followed by an SVM for classification.

Other feature-engineering approaches include [14], which combined Fast Fourier Transform (FFT), wavelet transform, and perceptual linear prediction, and [15], which proposed features based on spectral peak detection and band power ratios. A more recent approach, ref. [16], introduced a multi-scale active noise cancellation network (ANC-Net). Finally, illustrating the integration of physical principles, ref. [17] leveraged prior knowledge by integrating a diagnostic model with a machine learning technique for FD in a VSD-controlled induction motor.

Despite the high accuracy and ability to model complex fault signatures demonstrated by these data-driven and hybrid strategies, significant challenges remain for their widespread industrial adoption. Regarding deployment in real-world systems, while some studies have successfully implemented these algorithms on embedded platforms such as FPGAs [13] or high-performance microcontrollers [2] for real-time monitoring, the dominant workflow relies on offline training using extensive historical datasets. However, a common and critical limitation persists across most of the reviewed methods: their strong dependence on large amounts of labeled data representing both healthy and, most problematically, faulty conditions. As explicitly noted in [4], despite the good results reported, these methodologies require a “very large and diverse database.” Furthermore, ref. [5] emphasizes that acquiring such experimental data across different load conditions and failure levels is “extremely arduous in practical terms.”

In practice, industrial environments rarely provide access to such comprehensive datasets, as motors are not deliberately operated in degraded states. Consequently, most data-driven methods are trained on data derived from laboratory test benches under controlled conditions. When transferred to dynamic industrial environments, the discrepancy in data distribution—a challenge technically known as domain shift—often leads to performance degradation [5,18]. This fundamental limitation highlights the pressing need for novel approaches that can reduce dependency on faulty data and establish stronger theoretical links between the extracted features and the underlying physical fault mechanisms.

Conversely, following the model-based paradigm, a substantial body of research has focused on model-based and specialized signal analysis strategies for FD [19,20,21,22,23,24,25,26,27,28,29,30,31,32,33,34,35]. A prominent strategy involves residual analysis, where deviations between measured signals and a model-based observer are monitored. In this domain, recent theoretical frameworks have formalized the Residual Information Value (RIV) [19] as a rigorous statistical metric to quantify these deviations based on information theory, characterizing faults as model drifts. In the specific context of induction machines, for instance, ref. [20] proposes a residual spectrum analysis based on a state-space model, while digital twin approaches [21] and other methods [22] also calculate fault indices from this discrepancy. This concept is central to FD within advanced control frameworks like Model Predictive Control (MPC) [23,24]. However, a critical limitation of these methods is their high dependency on accurate model parameters ($R_{s},\;R_{r},\;L_{m}$, etc.), often requiring full motor knowledge [23], complex online identification routines [25], or specialized estimators like MRAS [26].

Alongside these observer-based methods, specialized signal-processing techniques focus on extracting specific fault signatures derived from physical principles. This includes classical harmonic analysis via FFT [27], time-frequency analysis using discrete wavelet transforms [28], and the analysis of sequence components [29,30]. Other strategies analyze specific control-related signals, such as PWM-induced common-mode voltage (CMV) sidebands [31] or internal signals from the DFOC controller [32]. While often effective, these methods can be sensitive to operational variability and may require deep domain expertise to select and interpret the relevant signal features.

However, a significant practical limitation shared by many of these approaches is their reliance on additional or non-standard sensors. For example, some methods require dedicated magnetic flux sensors mounted on the machine [33,34], which are rarely available in industrial setups. Others employ external antennas to measure radiated electromagnetic fields [35], dedicated torque sensors [24], or additional voltage sensors [30]. Even methods integrated into VSDs may require feedback from encoders [23,32] or current/voltage measurements at multiple points [29], increasing system cost, complexity, and potential points of failure.

These works, while demonstrating high sensitivity, highlight a clear trade-off: detection robustness is often achieved at the cost of requiring additional sensors [24,30,33,35] or precise, detailed motor parameters [20,23,26]. Both of these requirements represent significant barriers to practical adoption in diverse industrial environments.

In light of these discussed limitations—specifically, the dependency on extensive fault data in data-driven methods and the need for additional sensors or precise parameters in model-based approaches—this work introduces a novel fault detection framework based on the RIV principle, specifically targeting the challenging scenario of closed-loop operation where fault signatures are attenuated by the controller. By rigorously quantifying the information shared between the supply voltages and the current residuals, the proposed method detects faults as statistical model drifts, addressing the data scarcity and sensor cost barriers simultaneously. The main contributions are summarized as follows:

1. A Novel RIV-Based Fault Detection and Phase Discrimination Framework: We propose a diagnostic scheme that adapts the theoretical RIV concept [19] for induction machines. This method estimates the mutual information between the input vector (stator voltages) and the residual vector (the difference between measured and estimated stator currents). By characterizing faults as statistical changes in this dependency, the framework achieves robust detection and identifies the faulty phase without assuming specific fault distributions or requiring labeled fault data.
2. Experimental Validation of an Industry-Ready Strategy: The proposed framework is rigorously validated on a laboratory test bench driven by a standard industrial VSD. Addressing the implementation gap identified in the literature, this validation demonstrates the method’s applicability to realistic industrial scenarios by utilizing a self-commissioning routine for the nominal model generation. This approach allows for the diagnostic system to be deployed on loaded motors without mechanical decoupling or offline tests, bridging the gap between the theoretical RIV concept and practical, non-intrusive industrial implementation.

The remainder of this paper is organized as follows. Section 2 presents the theoretical background, including the motor modeling and sensorless control framework, the mutual information formulation, and the model drift–based fault detection concept. Section 3 details the proposed methodology and its integration into a unified monitoring architecture. Section 4 reports the experimental validation on an industrial VSD platform, and Section 5 concludes the paper with final remarks and perspectives for future work.

## 2. Background

### 2.1. Fault Detection as Statistical Model Drift

The recent work of Ramírez et al. [19] establishes a rigorous connection between fault detection and the detection of model drift, i.e., deviations in the deterministic input-output relationship of a system. This framework provides a powerful, distribution-free tool for fault detection.

#### 2.1.1. Additive Noise Model (ANM) Formulation

Let $X\in R^{p}$ and $Y\in R^{q}$ denote the input and output variables of a system. An additive noise model (ANM) is defined as

(1) Y=η(X)+h(W),

where $\eta :R^{p}\to R^{q}$ is the deterministic model, *W* is a noise variable independent of *X*, and h(·) is the noise model. Under this structure, any change in the deterministic function η(·) are defined as model drifts. Since faults correspond to undesired alterations in the system’s physical dynamics, they can be equivalently characterized as the presence of model drift.

#### 2.1.2. Fault Detection as Hypothesis Testing

Let $\hat{\eta }\left(\cdot \right)$ be the nominal model of the system (whose data-driven construction is detailed later in Section 3). The core idea is to test whether the real-time system behavior, denoted by an unknown function $\tilde{\eta }\left(\cdot \right)$, matches the nominal model $\hat{\eta }\left(\cdot \right)$. This is formalized as the following hypothesis test:

(2) $$\begin{array}{cc} \; & H_{0}:\;\tilde{\eta }\equiv \hat{\eta }\;\left(\mathrm{Healthy}\;\mathrm{system}\right),\\ \; & H_{1}:\;\tilde{\eta }≢\hat{\eta }\;\left(\mathrm{Fault}\;\mathrm{present}\right). \end{array}$$

A fundamental result from [19] establishes a direct equivalence between this test and the statistical independence of the input and the residual. If we define the residual as $R=Y-\hat{\eta }\left(X\right)$, then:

(3) $$H_{0}\;\Leftrightarrow \;X⊥\;\;\;⊥\;R,\;H_{1}\;\Leftrightarrow \;X⫫/\;R.$$

This equivalence holds because under $H_{0}$, the residual R=h(W) is just the system noise, which is independent of the input *X*. Conversely, under $H_{1}$, the residual contains a model-mismatch term $\left(\tilde{\eta }\left(X\right)-\hat{\eta }\left(X\right)\right)$, which makes *R* statistically dependent on *X*.

Therefore, the complex engineering problem of fault detection is rigorously transformed into a statistical problem: testing for independence between the input *X* and the residual *R*. To operationalize this theoretical test, we adopt the RIV framework. In the specific context of induction motor diagnostics, as established in the subsequent sections, the input variable *X* corresponds to the stator voltage vector and the output variable *Y* to the stator current vector.

### 2.2. The Residual Information Value (RIV) Detector

To implement the hypothesis test in (3), we adopt the RIV method introduced in [19]. The RIV detector operationalizes this test by using the mutual information (MI) as its test statistic, since I(X,R)=0 if and only if *X* and *R* are independent.

The RIV detection procedure, applied to a batch of *n* new samples, is as follows:

1. Collect data: Acquire *n* real-time input-output samples $Z_{n}={\left\{\left(X_{j},Y_{j}\right)\right\}}_{j=1}^{n}$ from the drive.
2. Compute residuals: Calculate the residuals using the nominal model $\hat{\eta }$ (detailed in Section 2.4):
  (4) $$R_{j}=Y_{j}-\hat{\eta }\left(X_{j}\right),\;j=1,\ldots ,n.$$
3. Estimate MI: Compute the MI value ${\hat{I}}_{n}\left(X;R\right)$ using the distribution-free estimator described in Section 2.3 on the sample set ${\left\{\left(X_{j},R_{j}\right)\right\}}_{j=1}^{n}$.
4. Make decision: Compare the estimated MI to a predefined threshold $\tau_{d}$, which determines the system’s False Alarm Rate (FAR):
  (5) $$\psi_{n}=\left\{\begin{array}{cc} 0, & {\hat{I}}_{n}\left(X;R\right)<\tau_{d}\;(\mathrm{Accept}\;H_{0}:\;\mathrm{system}\;\mathrm{healthy}),\\ 1, & {\hat{I}}_{n}\left(X;R\right)\geq \tau_{d}\;(\mathrm{Reject}\;H_{0}:\;\mathrm{fault}\;\mathrm{detected}). \end{array}\right.$$

This methodology is supported by strong theoretical guarantees [19]. These include strong consistency (the detector almost surely converges to the correct decision as n→∞) and exponentially fast detection bounds (the probability of a false alarm decays exponentially with the sample size *n*, while the detection power tends to one).

The RIV methodology provides a robust and unsupervised fault detection strategy. It does not require examples of faulty data, is agnostic to the system’s probability distribution (admitting non-linear and non-Gaussian cases), and can be applied using only the nominal model. These properties make it especially attractive for real-world industrial setups, such as induction motors, where failures often manifest as subtle model drifts and labeled faulty data are scarce or impossible to generate without damaging the equipment. However, the practical implementation of Step 3 requires a specialized estimator capable of handling these unknown distributions, which is presented next.

### 2.3. Distribution-Free Mutual Information Estimation

To implement the RIV detector described in Section 2.2, it is necessary to quantify the shared information between the inputs and residuals. Mutual information (MI), originally introduced by Shannon and Weaver, is the ideal metric for this task as it measures the reduction in uncertainty of one variable when another is known, capturing both linear and nonlinear statistical dependencies.

Formally, for two random variables *X* and *R*, MI is defined as:

(6) $$I\left(X,R\right)=\sum_{x\in X}\sum_{r\in R}p\left(x,r\right)\log\frac{p(x,r)}{p(x)\cdot p(r)},$$

where p(x) and p(r) are the marginal probability functions, and p(x,r) is the joint probability.

However, in real-time motor monitoring, the underlying probability distributions are unknown and non-Gaussian. Therefore, we employ the non-parametric and distribution-free estimator presented in [19,36]. Unlike computationally expensive kernel-based methods, this estimator computes ${\hat{I}}_{n}\left(X;R\right)$ from an i.i.d. sample $J_{n}={\left\{\left(X_{i},R_{i}\right)\right\}}_{i=1}^{n}$ without assuming any specific distributional form, operating in three key stages optimized for online efficiency:

Stage 1: Data-Driven Partitioning. The estimator constructs a partition $A={\left\{A_{\ell }\right\}}_{\ell \in L}$ of the joint space $R^{p+q}$ using a complexity-regularized binary tree. The space is recursively split by axis-parallel hyperplanes until each cell $A_{\ell }$ contains at most $n\cdot b_{n}$ data points, where $b_{n}$ controls the minimum cell mass. The tree is subsequently pruned using a regularization parameter λ>0 to prevent overfitting, ensuring a compact model representation suitable for real-time processing.
Stage 2: Empirical Probability Estimation. For each resulting cell $A_{\ell }=A_{\ell }^{\left(1\right)}\times A_{\ell }^{\left(2\right)}$, the empirical probabilities are computed as:
  (7) $$P_{n}\left(A_{\ell }\right)=\frac{1}{n}\sum_{i=1}^{n}{⊮}_{A_{\ell }}\left(X_{i},R_{i}\right),$$
  where ${⊮}_{A_{\ell }}$ is the indicator function. The marginal probabilities $P_{n}\left(A_{\ell }^{\left(1\right)}\times R^{q}\right)$ and $P_{n}\left(R^{p}\times A_{\ell }^{\left(2\right)}\right)$ are computed analogously.
Stage 3: MI Calculation. Finally, the mutual information is estimated as:
  (8) $${\hat{I}}_{n}\left(X;R\right)=\sum_{A_{\ell }\in A}P_{n}\left(A_{\ell }\right)\cdot \log\left(\frac{P_{n}\left(A_{\ell }\right)}{Q_{n}\left(A_{\ell }\right)}\right),$$
  where $Q_{n}\left(A_{\ell }\right)=P_{n}\left(A_{\ell }^{\left(1\right)}\right)\cdot P_{n}\left(A_{\ell }^{\left(2\right)}\right)$ represents the probability under the independence assumption. This estimator is consistent given appropriate parameter choices ($b_{n}\approx n^{-l},l\in \left(0,1/3\right)$), providing a reliable statistic for the RIV test.

### 2.4. Data-Driven Nominal Modeling and Sensorless Control

The foundation of the proposed RIV framework is a precise nominal model of the healthy motor. To construct this model without disassembling the drive or relying on often-unavailable manufacturer datasheets, we leverage the online self-commissioning algorithm presented in [37].

#### 2.4.1. Self-Commissioning for Parameter and Data Acquisition

This method is pivotal, as it estimates the set of induction motor and load parameters in approximately 180 s without disconnecting the motor from its load. Unlike traditional offline techniques, this approach employs a discrete normalized MRAS implemented on the TMS320F28388D microcontroller.

**Figure 1:**
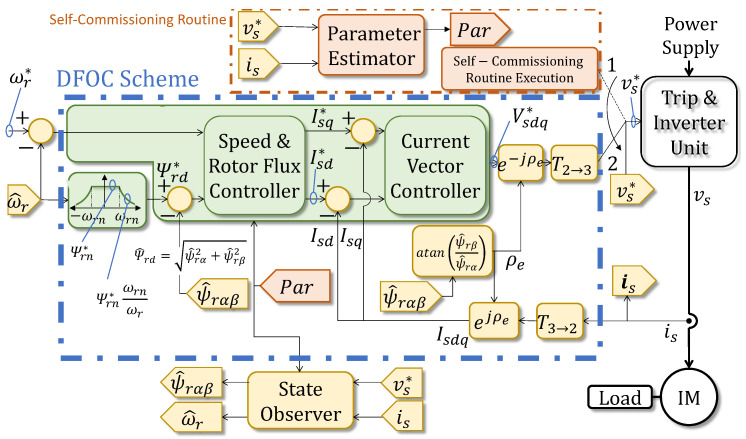
Block diagram of the self-commissioning and the Sensorless DFOC. The orange dashed area indicates the self-commissioning routine, while the blue dashed area represents the standard DFOC scheme. Superscripts with an asterisk (*) denote reference values.

As illustrated in the dashed block labeled “Self-Commissioning Routine” at the top of Figure 1, which directly references the offline identification workflow detailed in Section 3, this routine serves a dual purpose. First, the “Parameter Estimator” block calculates the estimated parameters (${\hat{R}}_{s},{\hat{L}}_{s},\sigma {\hat{L}}_{s}$, ${\hat{\tau }}_{r}$ and $\hat{J}$), which are collectively denoted by the tag Par and automatically injected into the control loops and observers for normal operation (switch position 2). Second, and crucially for this work, the rich time-series data (voltages $v_{s}^{*}$ and currents $i_{s}$) collected during this process (executed in switch position 1) provides the training dataset for the nominal model, establishing the healthy baseline $\hat{\eta }$ against which model drift is later quantified.

#### 2.4.2. Integrated Control Architecture

**Figure 2:**
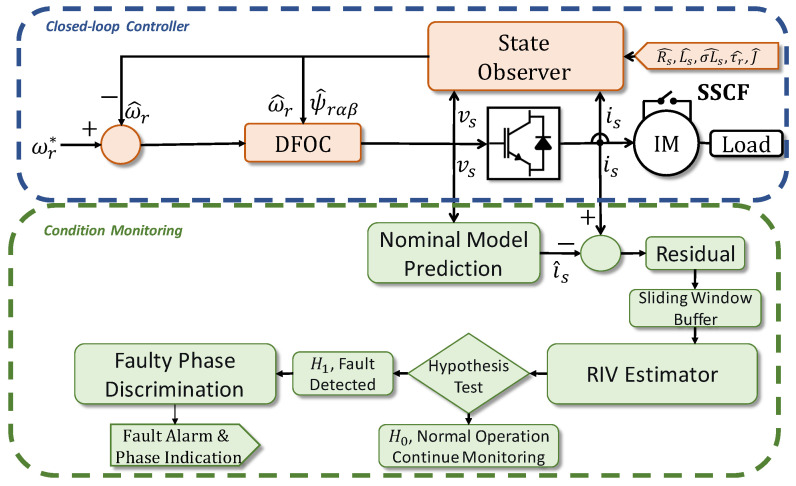
Online monitoring and diagnosis framework based on RIV detection. Superscripts with an asterisk (*) denote reference values.

Once the parameters are identified, they are deployed within a Sensorless DFOC architecture, encapsulated by the blue dashed box labeled “Sensorless DFOC Scheme” in Figure 1. This control strategy, which operates when the switch is in position 2, is selected for its high dynamic performance and elimination of mechanical sensors [8]. The control structure, depicted in the central green block within this scheme, uses a cascade arrangement with outer loops for speed/flux regulation and an inner loop for current control. To enable sensorless operation, a State Observer (specifically an Extended Kalman Filter) estimates the rotor speed ${\hat{\omega }}_{r}$ and flux ${\hat{\psi }}_{r\alpha \beta }$. As shown by the connection tag Par in Figure 1, this observer relies directly on the values (${\hat{R}}_{s},{\hat{L}}_{s}$, etc.) obtained from the self-commissioning stage. It is worth noting that this complete sensorless topology corresponds to the functional block simply labeled “DFOC” in the monitoring architecture of Figure 2. The complete integrated system provides a stable and known operational context, ensuring that the RIV detector monitors the motor health under controlled conditions.

### 2.5. Baseline Methods and Performance Metrics

To validate the proposed RIV framework, a comparative analysis is conducted against established diagnostic techniques. This section defines the physical principles and operational limitations of these baseline methods.

#### 2.5.1. Spectral Signature Analysis

Motor Current Signature Analysis (MCSA) remains the industrial standard for induction motor diagnosis due to its non-intrusive nature [4]. This method relies on the detection of characteristic harmonic sidebands in the stator current spectrum induced by the magnetic asymmetry of the fault. For inter-turn short circuits (ITSC), the characteristic fault frequencies ($f_{fault}$) are typically located at [15,32]:

(9) $$f_{fault}=f_{s}\left(1\pm k\frac{n}{p}\left(1-s\right)\right)$$

where $f_{s}$ is the supply frequency, *s* is the slip, *p* is the pole pair number, and k,n are integers.

While effective in stationary regimes, MCSA faces a fundamental physical trade-off between frequency resolution (Δf) and detection latency. To distinguish fault sidebands that are close to the fundamental frequency (i.e., small Δf), the FFT requires a data acquisition window $T_{w}$ inversely proportional to the desired resolution ($T_{w}=1/\Delta f$). Consequently, obtaining high spectral resolution inevitably introduces a significant detection delay. Furthermore, as noted in [33], for incipient faults or low-severity short circuits, the amplitude variations in these sidebands are often negligible (see Figure 4 in [33]), rendering the method insensitive to early-stage failures and susceptible to false negatives. However, the sensitivity of MCSA strongly depends on the selected time-window length and signal-to-noise ratio, which limits its effectiveness for incipient faults.

#### 2.5.2. Residual Energy Monitoring (Model-Based Baseline)

To rigorously benchmark the proposed method against model-based approaches, we implemented the residual energy monitor following the stationary frame formalism described in Digital Twin frameworks [21]. The detection logic proceeds in three steps:

First, the instantaneous residual vector $r_{abc}\left(t\right)$ is computed as the difference between the measured stator currents and the nominal model predictions ($r_{abc}=i_{s}-{\hat{i}}_{s}$).

Second, the three-phase residuals are projected onto the stationary two-phase reference frame (αβ). This transformation aligns the diagnostic signals with the vector control variables, facilitating the analysis of the residual space vector magnitude.

Third, the Fault Detection Index (FDI) is calculated as the instantaneous energy of the stationary residual vector. This metric, denoted here as E(t), captures the total deviation of the current space vector from its expected trajectory:

(10) $$E\left(t\right)=r_{\alpha }^{2}\left(t\right)+r_{\beta }^{2}\left(t\right)$$

Finally, the detection algorithm compares this energy index against a statistical threshold $E_{th}$, which is calibrated based on the peak noise observed during healthy operation (conventionally set at 3σ of the healthy baseline noise):

(11) $$E\left(t\right)>E_{th}\Rightarrow \mathrm{Fault}\;\mathrm{Detected}$$

While theoretically sound, the reliance on the magnitude of the residual vector makes this method highly sensitive to transient modeling errors. As will be shown in the Section 4, rapid load or speed changes cause momentary spikes in E(t) that can be misclassified as faults if the threshold is not set conservatively high.

#### 2.5.3. Quantitative Performance Metrics

To objectively evaluate the proposed method against the baselines and address the need for rigorous validation, we adopt three quantitative metrics. These metrics are grounded in the theoretical performance analysis for hypothesis testing established in [19]:

1. Detection Delay ($T_{d}$): Formally related to the finite-sample collapsing time defined in [19], this metric measures the time elapsed between the physical fault inception ($t_{fault}$) and the moment the decision rule $\psi_{n}$ stabilizes on the rejection of $H_{0}$:
  (12) $$T_{d}=t_{alarm}-t_{fault}$$
  where $t_{alarm}=\min\left\{t>t_{fault}:\psi_{n}\left(t\right)=1\right\}$. Comparison of $T_{d}$ highlights the trade-off between the fast statistical convergence of RIV versus the window-dependent latency of MCSA.
2. False Alarm Rate (FAR): This metric corresponds to the empirical significance level ($\alpha_{n}$) of the test [19]. It quantifies the robustness of the detector against dynamic transients (e.g., load impacts) when the motor is healthy:
  (13) $$\mathrm{FAR}=P\left(\psi_{n}=1|H_{0}\right)\approx \frac{N_{false}}{N_{healthy}}$$
  where $N_{false}$ is the number of samples where a fault is incorrectly declared during healthy operation.
3. Separability Index (Fisher Score F): To quantify the discriminative power of the fault indicator beyond simple thresholding, we utilize the Fisher Score. This index measures the statistical distance between the healthy distribution ($\mu_{0},\sigma_{0}^{2}$) and the faulty distribution ($\mu_{1},\sigma_{1}^{2}$) of the diagnostic index:
  (14) $$F=\frac{{\left(\mu_{1}-\mu_{0}\right)}^{2}}{\sigma_{1}^{2}+\sigma_{0}^{2}}$$
  A higher F value indicates superior fault sensitivity and a lower probability of classification error.

With the theoretical foundations, baseline comparison methods, and quantitative evaluation metrics fully defined, the subsequent section presents the experimental validation of the proposed framework.

## 3. Proposed Methodology

This section describes the proposed fault detection and phase discrimination methodology, which integrates a data-driven nominal model, residual generation, and RIV estimation into a unified online monitoring architecture.

To address the limitations identified in Section 1, this work proposes a fault detection and diagnosis framework grounded in the RIV principle. Unlike traditional methods that rely on labeled fault data or intrusive signal injection, our approach leverages the statistical concept of model drift to identify incipient stator faults using only standard control signals. The proposed strategy is integrated directly into the drive’s control architecture, enabling reliable operation under realistic industrial conditions.

### Integrated RIV-Based Monitoring Scheme

The core of the proposed methodology is the real-time monitoring loop illustrated in Figure 2. To visually distinguish the novel contributions of this work, the diagnostic functional blocks are highlighted in green. This distinction also corresponds to the hardware architecture: the standard control loops (blue blocks) are executed on the embedded DSP to guarantee real-time stability, while the diagnostic algorithms (green blocks) are hosted on an external monitoring station (e.g., an industrial PC). This decoupled design ensures that the statistical analysis does not burden the control processor and facilitates the method’s extension to other VSDs capable of streaming $v_{sabc}$ and $i_{sabc}$ data. This system operates in parallel with the control structure, passively processing the input voltages and output currents to detect statistical anomalies without affecting the controller performance. The framework is composed of four sequential stages: nominal model prediction, residual generation, RIV estimation, and faulty phase discrimination.

**Figure 3:**
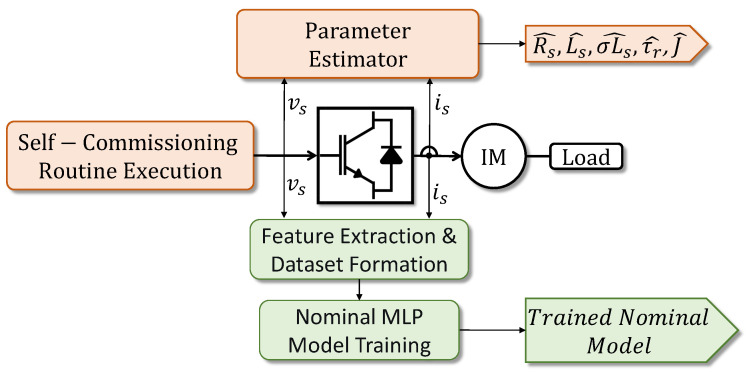
Workflow for the offline construction of the data-driven nominal model from self-commissioning data. The orange blocks represent the standard self-commissioning routine, while the green blocks indicate the proposed data-driven modeling stages.

The process begins at the Nominal Model Prediction block. Here, the real-time voltage vector X(k) is fed into a pre-trained nominal model $\hat{\eta }$ to estimate the expected healthy currents $\hat{Y}\left(k\right)$. Crucially, the proposed framework operates in the natural abc reference frame rather than the synchronous dq frame. This design choice is strategic: unlike the dq transformation which distributes a single phase fault across both axes, the abc representation preserves the unique spatial signature of each winding. This feature is essential for the direct Faulty Phase Discrimination stage (detailed later) and simplifies the neural model structure by removing the dependency on the rotor flux angle for synchronization. The construction of this specific block is a critical component of the methodology. To ensure the model $\hat{\eta }$ accurately represents the healthy baseline without requiring manual data collection, we utilize the data extracted from the self-commissioning routine described in [37] and depicted in Figure 3. The raw waveforms ($v_{sabc},i_{sabc}$) collected during this 180-s initialization process cover the entire dynamic excitation range of the motor. This dataset is used to train the mapping from voltage history to current response.

It is important to emphasize that the proposed RIV framework is model-agnostic; the nominal model $\hat{\eta }$ could be implemented using analytical physics-based equations, RNNs, or other regression tools. However, in this implementation, an MLP is selected specifically for its structural simplicity and low computational burden. The MLP offers sufficient approximation capacity to ensure that the residuals are uncorrelated with inputs under healthy conditions, which is the only requirement for the RIV test to function correctly. Thus, the model $\hat{\eta }$ deployed in this block is essentially a data-driven, open-loop observer of the healthy stator impedance.

**Remark 1.**  *It is assumed that the motor operates under healthy or near-healthy conditions during the self-commissioning procedure used to train the MLP. If the motor already exhibits significant stator degradation at this stage, the resulting nominal model may embed these faulty dynamics, potentially masking subsequent fault detection.*

Following the prediction, the Residual Generation stage computes the instantaneous deviation between the measured currents Y(k) and the model estimates $\hat{Y}\left(k\right)$. The residual vector is defined as:

(15) $$R\left(k\right)=Y\left(k\right)-\hat{Y}\left(k\right)={\left[i_{sa}-{\hat{i}}_{sa},\;i_{sb}-{\hat{i}}_{sb},\;i_{sc}-{\hat{i}}_{sc}\right]}^{T}.$$

The use of current residuals is advantageous as these variables are already measured by the VSD for control purposes, eliminating the need for additional hardware such as vibration sensors or flux probes.

These residuals are accumulated in a sliding window buffer to form the dataset necessary for statistical analysis. The RIV Estimator block then acts as the core engine of the method. It computes the statistical dependency $\hat{I}\left(X;R\right)$ between the voltage inputs and the current residuals using the distribution-free estimator described in Section 2.3. This step operationalizes the RIV test: under healthy conditions, the residuals should be independent of the inputs (noise); under a fault, the physical modification of the machine creates a detectable dependency ($\hat{I}>0$).

The decision logic compares the estimated RIV metric $\hat{I}$ against a threshold $\tau_{d}$. This threshold is calibrated empirically using the statistics of the RIV distribution observed during a brief healthy initialization period. If $\hat{I}$ exceeds $\tau_{d}$, the system triggers the Faulty Phase Discrimination stage.

Instead of complex pattern recognition, this stage leverages the localized nature of the RIV to identify the fault location. We compute three separate, per-phase mutual information values:

(16) $$\begin{array}{cc} {\hat{I}}_{a} & =\hat{I}\left(X,R_{a}\right)=\hat{I}\left(X,i_{sa}-{\hat{i}}_{sa}\right) \end{array}$$

(17) $$\begin{array}{cc} {\hat{I}}_{b} & =\hat{I}\left(X,R_{b}\right)=\hat{I}\left(X,i_{sb}-{\hat{i}}_{sb}\right) \end{array}$$

(18) $$\begin{array}{cc} {\hat{I}}_{c} & =\hat{I}\left(X,R_{c}\right)=\hat{I}\left(X,i_{sc}-{\hat{i}}_{sc}\right) \end{array}$$

The global fault indicator $\hat{I}$ can be defined as their aggregation (e.g., maximum or sum). Once a fault is declared, the faulty phase $P_{fault}$ is identified by finding the phase that contributes the most to this statistical dependency:

(19) $$P_{fault}=\underset{p\in \{a,b,c\}}{\arg\max}\left({\hat{I}}_{p}\right)$$

This identification principle relies on the physical premise that a short circuit induces the strongest model drift—and consequently the highest statistical dependency—in its corresponding phase residual.

It is important to note that this monitoring scheme is fully integrated with the Sensorless DFOC architecture shown in Figure 1. The control loops and the Extended Kalman Filter (EKF) observer [37] continue to regulate the machine using the parameters identified during self-commissioning, while the RIV framework operates in the background. This ensures that the diagnosis is performed within a stable, controlled context.

## 4. Experimental Results

### 4.1. Experimental Setup and Test Description

To validate the proposed framework, extensive experiments were conducted on a laboratory test bench under realistic operating conditions. The objective of this evaluation is to rigorously assess the detection sensitivity, response time, and phase discrimination capability of the proposed method when addressing SSCF. All tests were executed under controlled and repeatable conditions to ensure the reliability of the reported results. To mitigate the effects of thermal degradation on the stator winding resistance during the experimental campaign, the test duration was kept short (approx. 15 s per trial), and a cool-down period of 20 min was enforced between consecutive experiments to allow the machine to return to its ambient thermal equilibrium.

**Figure 4:**
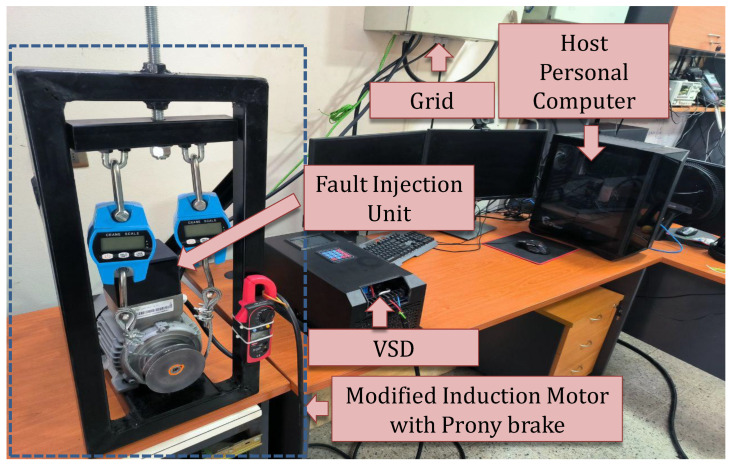
Experimental test bench setup comprising the custom-designed VSD and the modified Siemens induction motor.

Figure 4 illustrates the laboratory test bench employed for the experimental validation. The setup relies on a Siemens induction motor (model 1LE0101-0DA22-2AA4), structurally modified to emulate stator faults. The detailed technical specifications of this machine are listed in Table 1. The motor is driven by a custom-designed Variable Speed Drive (VSD) developed at the university laboratory, which utilizes a Texas Instruments TMS320F28388D DSP to command a two-level voltage-source inverter. Regarding the software implementation, MATLAB R2024a and Simulink 24.1 were used to design, compile, and deploy the discrete-time control algorithms onto the VSD’s embedded processor. During operation, the DSP streams voltage and current measurements to the host computer via a high-speed serial interface. The diagnostic algorithm, running in parallel on the host, processes the sliding window buffer sequentially. Since the execution time of the RIV estimator and MLP inference is negligible compared to the data acquisition period, the setup operates as a real-time monitoring system, providing instantaneous fault feedback.

All diagnostic signals employed in this study are directly acquired from the drive’s internal measurements, without the need for additional sensors or hardware modifications.

During the experimental tests, the induction machine operated at a mechanical speed of approximately 1000 rpm. The motor was mechanically loaded using a Prony brake system, as visible in Figure 4. This mechanism applies a frictional load to the motor shaft, which is manually regulated via an adjustment handle to establish the desired torque operating point.

**Figure 5:**
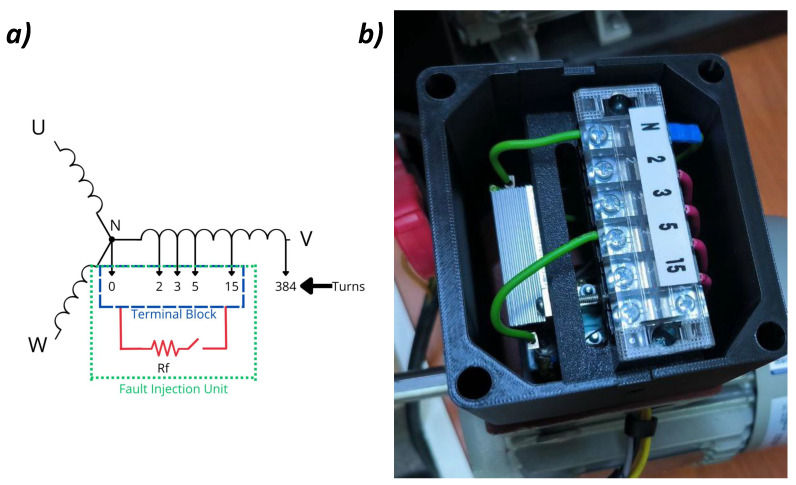
SSCF emulation: (**a**) electrical schematic illustrating the external resistor connection to the winding taps and (**b**) photograph of the physical implementation on the test motor.

To validate the proposed method, Stator Short-Circuit Faults (SSCF) were physically emulated on the test motor. As illustrated in Figure 5, this was achieved by connecting calibrated external resistors between dedicated taps in the stator winding (specifically in Phase B). Figure 5a shows the electrical schematic of this connection, while Figure 5b provides a photograph of the modified motor terminals. This approach enables the controlled and repeatable replication of incipient faults while preserving the electromagnetic behavior of true insulation degradation. The fault severity is defined by the percentage of shorted turns relative to the total turns in the phase; severities ranging from 1% to 4% were tested. Although the fault-emulation taps were installed exclusively on Phase B due to manufacturing constraints, the symmetrical nature of the induction machine ensures that these results are representative of faults occurring in any of the three phases.

Finally, to ensure the reproducibility of the proposed data-driven framework, the specific hyperparameters configured for both the Nominal Model (MLP) and the RIV Estimator are detailed in Table 2.

Regarding the MLP, the input size corresponds to the time-delay structure defined in the Section 3 (15 inputs). Although the proposed RIV framework is model-agnostic, an MLP was selected here for its computational efficiency. The choice of a single hidden layer is grounded in the Universal Approximation Theorem [38], which posits that this structure is sufficient to represent continuous motor dynamics. Furthermore, the specific size of 64 neurons was determined via an empirical grid search to minimize the validation error, prioritizing a compact model to prevent overfitting. For the RIV estimator, the parameters ($\lambda ,b_{n}$) were tuned based on the consistency conditions [19] where different values were tested to ensure convergence, specifically setting the cell mass decay exponent to 0.167. As shown in the table, three different sliding window lengths (n∈{100,180,250}) were evaluated, with n=180 selected as the optimal operating point for the reported results.

To validate the performance against established methods, two baselines were configured following standard literature guidelines. The MCSA method was implemented using a Hanning window of $T_{w}=1.0$ s to ensure sufficient frequency resolution for sideband detection as recommended in [15,32]. Simultaneously, the Residual Energy monitor was set up according to the digital twin principles described in [21], utilizing a fixed detection threshold set at 3σ of the steady-state noise observed during healthy operation.

### 4.2. RIV Calibration and Detection Results

**Figure 6:**
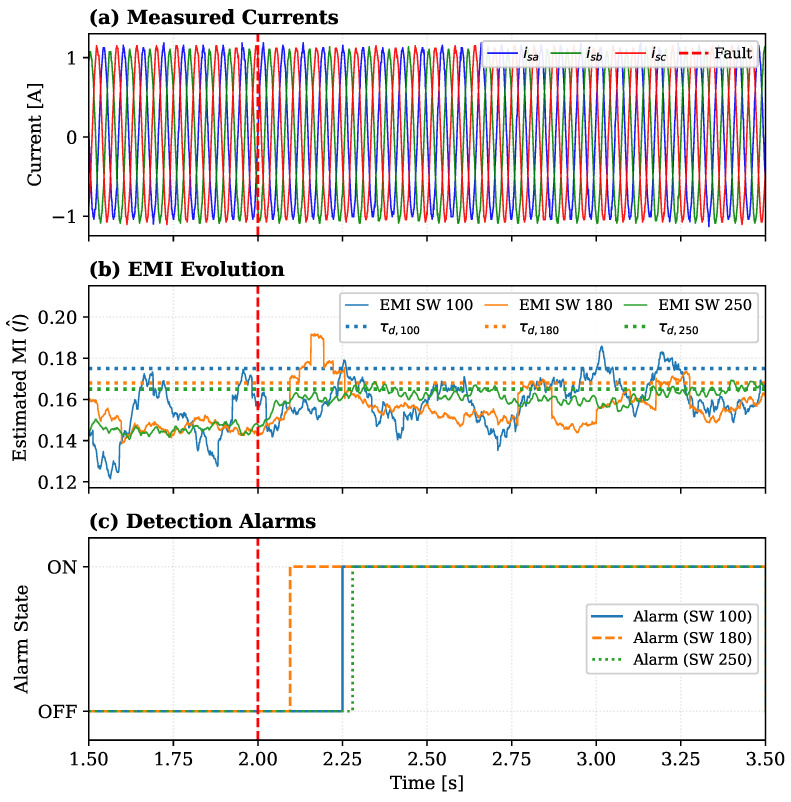
System response to a 1% stator short-circuit fault induced in Phase B at t=2s. The plots show: (**a**) measured three-phase currents ($i_{sa},i_{sb},i_{sc}$); (**b**) EMI evolution ($\hat{I}$) for three sliding window (SW) lengths with their respective thresholds ($\tau_{d}$); and (**c**) corresponding binary fault alarm signals. The vertical red dashed line indicates the fault inception time ($t_{fault}$).

This subsection evaluates the ability of the proposed framework to detect SSCF at its incipient stages and establishes the optimal hyperparameter configuration for the subsequent comparative analysis. Figure 6 reports the system’s response to a representative 1% inter-turn short-circuit fault induced in Phase B at $t_{fault}=2.0$ s. The figure simultaneously illustrates the detection sensitivity and the critical trade-off between detection speed and estimator noise, which is governed by the sliding window (SW) length.

The figure is organized into three plots. The top plot shows the measured three-phase stator currents ($i_{sa},i_{sb},i_{sc}$). Even after the fault inception at $t_{fault}$, the currents exhibit only a very subtle asymmetry, demonstrating the challenge of detecting such an incipient fault using simple current monitoring.

The middle plot shows the evolution of the estimated global mutual information, $\hat{I}$, for three different SW lengths (100, 180, and 250 samples). This plot clearly demonstrates the core of the proposed method:

- Healthy State (t<2.0 s): Before the fault, all three $\hat{I}$ estimators remain stable and fluctuate below their respective empirically-calibrated detection thresholds ($\tau_{d,100},\tau_{d,180},\tau_{d,250}$). This confirms the method’s robustness against false alarms during normal operation.
- Fault State (t>2.0 s): Immediately after the fault, the statistical dependency I(X;R) increases, causing all three estimators to rise and promptly cross their thresholds.

The bottom plot confirms this logic by showing the resulting binary fault alarms. Each alarm is triggered precisely when its corresponding estimator in the middle plot crosses its threshold. This visualization captures the fundamental trade-off of the window length:

- SW 100 (Blue): Although theoretically faster, it exhibits the highest variance (noise). As observed in the middle plot, this volatility can momentarily delay the threshold crossing, resulting in a detection time of 0.25 s in this trial.
- SW 180 (Orange): Provides the fastest detection in this scenario (95 ms) while maintaining a balanced compromise between response speed and signal stability.
- SW 250 (Green): Provides the smoothest signal (lowest noise) but results in the longest detection delay (approx. 0.28 s) due to the higher filtering effect.

These results confirm that the proposed framework successfully detects a 1% SSCF—a challenging incipient-level event—in under 0.3 s. Based on this analysis, a sliding window of 180 samples was selected as the optimal operating point for the subsequent tests.

The discrimination logic relies on the three separate MI estimators defined in Equations (10)–(12): ${\hat{I}}_{a}$, ${\hat{I}}_{b}$, and ${\hat{I}}_{c}$. The faulty phase $P_{fault}$ is identified by finding the estimator that shows the largest deviation from its nominal behavior, following the $\arg\max(I_{p})$ rule.

**Figure 7:**
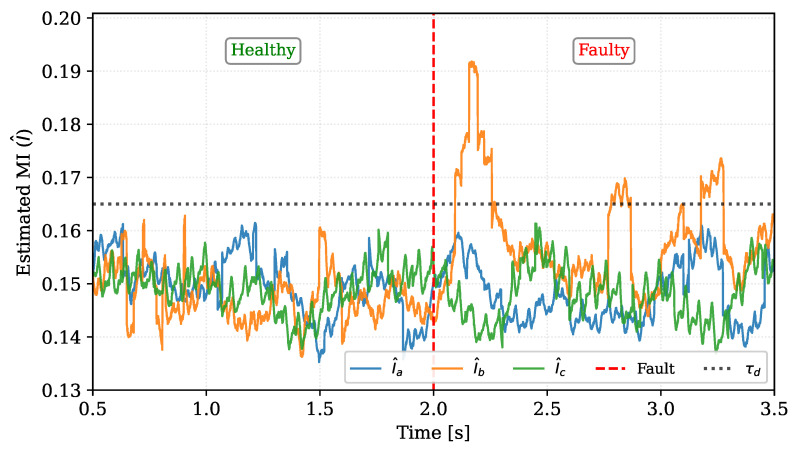
Evolution of the smoothed, per-phase MI estimators (${\hat{I}}_{a},{\hat{I}}_{b},{\hat{I}}_{c}$) during a 1% SSCF induced in Phase B at $t_{fault}$.

Figure 7 provides a clear validation of this mechanism for the 1% SSCF in Phase B (using the optimal SW 180 configuration). The plot shows the evolution of the three per-phase MI estimators, which naturally operate at different baseline offsets during healthy operation.

As shown in Figure 7, during the Healthy Operation phase (t<2.0 s), all three estimators fluctuate within their established nominal bands. Although the estimators exhibit inherent statistical variance (particularly Phase A), they remain bounded. However, immediately following the Faulty State inception, the estimator corresponding to the faulty phase (${\hat{I}}_{b}$, orange) exhibits a clear and sustained increase, breaking away from its healthy baseline. In contrast, the estimators for the healthy phases (${\hat{I}}_{a}$, blue, and ${\hat{I}}_{c}$, green) continue to fluctuate within their original ranges, showing no correlation with the fault event.

This result visually confirms that the model drift caused by the fault is almost exclusively captured by the MI estimator of the affected phase. This clear divergence allows the argmax rule to robustly and correctly identify the faulty phase. With the detection parameters calibrated and the discrimination capability verified, this configuration serves as the basis for the comparative benchmarking against the state-of-the-art methods presented next.

### 4.3. Performance of Baseline Methods

To establish a comparative benchmark for incipient fault detection, the experimental scenarios were first analyzed using the classical Motor Current Signature Analysis (MCSA) and the Model-Based Residual Energy method.

#### 4.3.1. Spectral Analysis Limitations (MCSA)

To evaluate the feasibility of classical spectral methods, the stator current ($I_{b}$) was analyzed using Fast Fourier Transform (FFT). The tests were conducted at the nominal speed of 1000 rpm ($f_{s}\approx 33.3$ Hz), ensuring the same experimental conditions used for the proposed RIV evaluation. Following the methodology in [32], the analysis focuses on the amplitude of the sideband component at $2f_{s}$, which is characteristic of inter-turn short-circuit faults.

**Figure 8:**
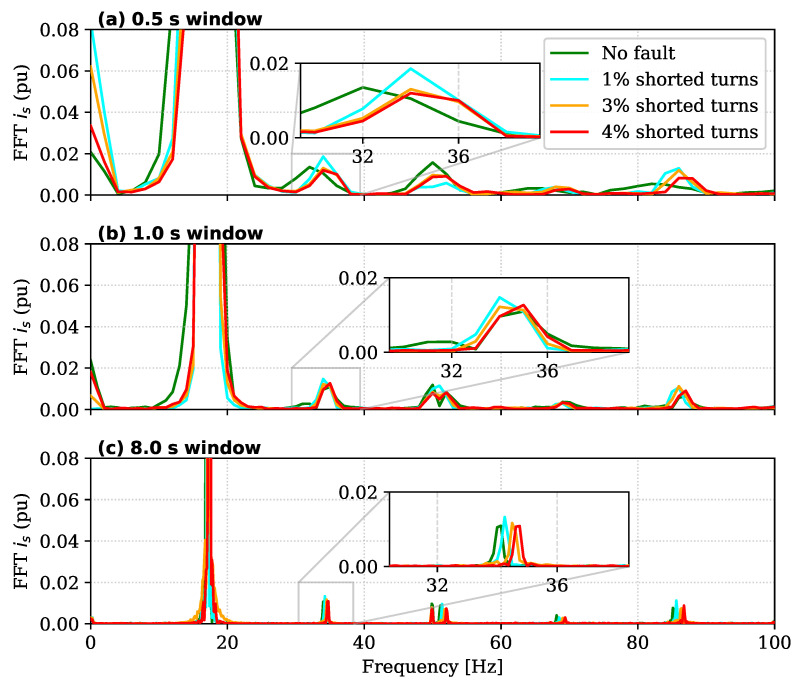
Impact of time-window length on MCSA sensitivity at 1000 rpm ($I_{b}$ spectrum): (**a**) 0.5 s window, (**b**) 1.0 s window, (**c**) 8.0 s window.

Figure 8 illustrates the impact of the acquisition window length ($T_{w}$) on the detectability of this fault signature. The results reveal a critical trade-off between frequency resolution and detection latency:

As observed in Figure 8a, applying a short time window of 0.5 s—desirable for fast detection—results in significant spectral leakage. The main lobe of the fundamental frequency widens considerably, masking the $2f_{s}$ fault harmonic. Extending the window to 1.0 s (Figure 8b) improves resolution but fails to isolate incipient faults; the 1% severity case (orange line) remains indistinguishable from the healthy baseline.

A clear separation of the fault harmonic is only achieved by increasing the window length to 8.0 s, as shown in Figure 8c. These results are consistent with those reported in [32], confirming that MCSA is effective for detecting incipient faults (1%, 3%, and 4%) provided that sufficient spectral resolution is available. However, the required acquisition time (>8 s) introduces a significant latency, making the method unsuitable for real-time protection against rapidly evolving faults.

#### 4.3.2. Residual Energy Sensitivity

To ensure a fair comparison with the proposed RIV method, the Residual Energy strategy was evaluated under identical experimental conditions, specifically at a constant speed of 1000 rpm. Implemented using the same MLP architecture described in Section 4.1, this approach aligns with standard digital twin methodologies [21]. As detailed in Table 2, a moving average filter ($N_{ma}=100$) was applied to the raw residuals to suppress high-frequency sensor noise. The detection threshold ($\epsilon_{th}$) was then empirically calibrated based on the filtered healthy baseline to minimize the False Alarm Rate (FAR), ensuring that normal noise fluctuations do not trigger spurious detections.

**Figure 9:**
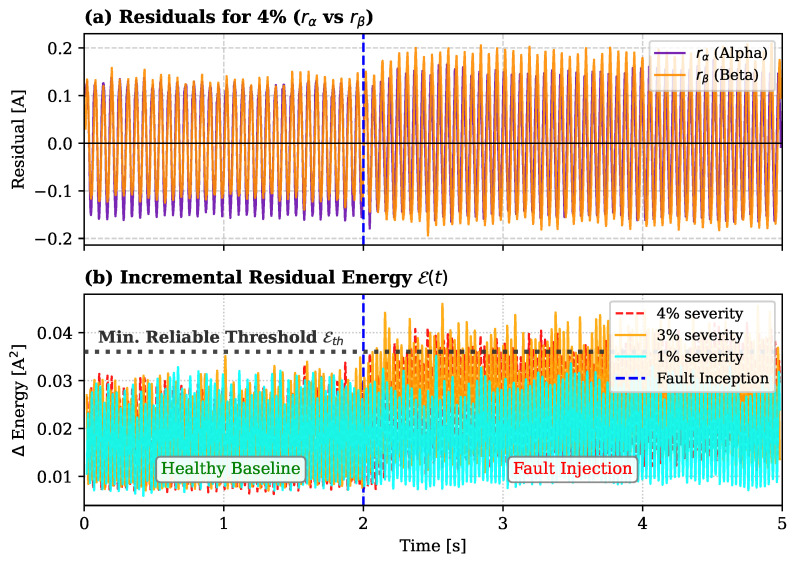
Residual-based detection performance. (**a**) Instantaneous residuals $\left(r_{\alpha },r_{\beta }\right)$ for 4% severity. (**b**) Incremental energy E(t) compared against the 3σ threshold. The solid black line in (**a**) marks the zero level, while the dashed horizontal line in (**b**) indicates the detection threshold ($E_{th}$).

The detection capabilities are visually confirmed in Figure 9a, where the instantaneous residuals for the 4% severity case exhibit a distinct deviation from zero after the fault injection. This clear signature translates into the energy domain shown in Figure 9b, where faults with severities of 3% and 4% rapidly exceed the threshold $\epsilon_{th}$ after t=2.0 s. In contrast, the 1% severity case (cyan trajectory) remains comparable to the noise floor, masked by the residual variance. This confirms that while energy-based metrics are effective for moderate faults, they lack the necessary sensitivity for incipient stages under realistic noise conditions.

MCSA and residual-energy-based detection were selected as baselines because they represent widely adopted spectral and model-based diagnostic strategies in industrial practice.

### 4.4. Overall Performance Comparison and Discussion

To conclude the experimental study, a comprehensive comparison is presented between the proposed RIV method and the baseline strategies (MCSA and Residual Energy) discussed in Section 4.3. This comparison is based on the key performance indicators defined in Section 2.5.3: detection delay, statistical separability (Fisher Score), and the False Alarm Rate (FAR). Additionally, the capability to correctly identify the affected phase is verified as a fundamental requirement for all diagnostic methods. The results for the different fault severities at 1000 rpm are summarized in Table 3.

The proposed RIV-based method consistently achieves lower detection delay and higher statistical separability than the baseline techniques, particularly for incipient fault conditions. The detection delay analysis reveals a clear advantage for the proposed RIV method. For the most critical case (1% severity), the RIV achieves a stable detection in only ≈61 ms. In contrast, the MCSA requires an acquisition window of at least 8.0 s to resolve the $2f_{s}$ harmonic from the fundamental spectral leakage, while the Residual Energy method fails to consistently exceed the threshold, rendering the incipient fault undetectable. This difference of two orders of magnitude in latency highlights the RIV’s suitability for near-instantaneous protection.

Regarding statistical separability, the Fisher Score (F) confirms the superiority of the information-based approach. The RIV maintains a significantly higher score (0.813) compared to the Residual Energy method (0.006) at 1% severity. This indicates that the RIV effectively extracts fault signatures that are otherwise masked by noise in the time-domain energy calculation. It should be noted that the Fisher Score is not applicable to MCSA, as the latter relies on frequency-domain snapshots rather than continuous time-series distributions.

The False Alarm Rate (FAR) was evaluated using a large healthy dataset characterized by frequent variations in speed and load to test the detectors under dynamic transients. The MCSA exhibits the lowest FAR, as its long-term averaging naturally filters out transient oscillations; however, this comes at the cost of high data requirements. Among the fast-response methods, the Residual Energy approach showed poor robustness with a high FAR (<25%), as load impacts were frequently misinterpreted as faults. The RIV method significantly mitigates this issue (<0.6%), providing a robust diagnostic even under varying operating conditions.

Finally, all three methods demonstrated the capability to correctly identify the faulty phase ($I_{b}$), confirming that the proposed RIV method preserves the fundamental diagnostic features of traditional baselines while significantly improving sensitivity and response speed.

## 5. Conclusions

This work presents a high-sensitivity fault detection framework based on the Residual Information Value (RIV) principle, specifically designed to address the data bottleneck in industrial diagnostics. By treating fault detection as a statistical test of independence between control signals and current residuals, the proposed method breaks a major industry obstacle: the heavy dependency on large, labeled fault datasets for training. The framework’s primary advantage lies in its seamless integration into standard industrial VSDs, leveraging existing self-commissioning routines to train a lightweight MLP nominal model without requiring additional sensors or intrusive signal injection.

Experimental results on an industrial-grade motor confirm that the RIV method substantially advances the state of the art. While traditional MCSA requires acquisition windows of at least 8.0 s to resolve incipient signatures, the RIV framework achieves reliable detection of 1% stator short-circuit faults in only 61 ms. Furthermore, the proposed method successfully overcomes the reliability issues of conventional residual-energy schemes: whereas standard energy indicators exhibit an unacceptable False Alarm Rate of up to 25% for incipient faults, the RIV approach reduces this metric to below 0.6%. This robustness is further supported by the Fisher Score, which reveals that the RIV extracts fault information that is over 130 times more statistically significant than time-domain energy indicators for 1% severity cases, providing a precise and model-agnostic tool for early protection.

Looking forward, several research directions emerge to further extend these contributions. Future studies will explore a hybrid diagnostic architecture by integrating the proposed statistical drift indicator with physical-model residuals derived from the DFOC’s internal Extended Kalman Filter (EKF). The deployment of the framework as a fully embedded edge-computing function within the VSD’s DSP constitutes a promising path for immediate industrial adoption. Because the underlying hypothesis is generic, future work will also investigate the applicability of this model-drift paradigm to other fault classes, including stator open-circuit faults, bearing degradation, broken rotor bars, and mechanical imbalances. Furthermore, since the methodology relies on fundamental input-output constitutive relations rather than specific machine parameters, it is theoretically transferable to other AC motor types, such as PMSM or SynRM, as long as the requisite signals are available. To further enhance robustness against long-term thermal variations, future iterations of the nominal model will incorporate real-time stator resistance estimates (${\hat{R}}_{s}$) as an auxiliary input to the MLP, ensuring the framework’s stability in diverse and demanding industrial environments.

## Figures and Tables

**Table 1:** Nameplate data and parameters of the induction machine under test.

Symbol [Unit]	Description	Value
$P_{n}$ [W]	Rated active power	750
$\sqrt{3}V_{sn}$ [V]	Rated line-to-line voltage	400
$I_{sn}$ [A]	Rated phase current	1.67
${\mathrm{PF}}_{n}$	Rated power factor	0.84
$f_{n}$ [Hz]	Rated electrical frequency	50
*p*	Number of pole pairs	1
$\omega_{rn}$ [rad/s]	Rated rotor angular speed	292.69
$\omega_{en}=2\pi f$ [rad/s]	Rated angular electrical speed	314
$T_{n}=\frac{P_{n}}{\omega_{rn}}$ [Nm]	Rated torque	2.56
$J_{m}$ [10^−3^ kg m^2^]	Motor inertia	1.6

**Table 2:** Configuration of the Diagnostic Algorithms.

Algorithm	Parameter	Value/Setting
Nominal MLP	Input Layer Nodes	15 (Voltage History)
	Hidden Layer Nodes	64
	Output Layer Nodes	3 (Stator Currents)
	Optimization	Adam (α=0.001)
	Loss Function	MSE (Mean Squared Error)
Proposed RIV	Sliding Window (*n*)	Tested: {100, 180, 250} → **180**
	Regularization (λ)	$2\times 10^{-5}$
	Min. Cell Mass ($b_{n}$)	$0.05\cdot n^{-0.167}$
MCSA (Baseline)	Spectral Window	Hanning ($T_{w}=1.0$ s)
Residual Energy	Detection Threshold	Empirically tuned ($J_{th}=0.036$)
	Noise Suppression	Moving Average ($N_{ma}=100$)

*Note:* The bold value indicates the selected configuration.

**Table 3:** Comparative Performance Analysis across Different Fault Severities at 1000 RPM.

Metric	Severity	MCSA	Residual Energy	Proposed RIV
Detection Delay	4%	≥1.0 s	≈220 ms	≈61 ms
	3%	≥1.0 s	≈94 ms	≈90 ms
	1%	≥8.0 s	Not Detected	≈95 ms
Fisher Score (F)	4%		0.450	5.66
	3%	N/A	0.425	3.29
	1%		0.006	0.813
False Alarm Rate (FAR)	Healthy	Very Low	Moderate (<25%)	Very Low (<0.6%)
Phase Detection	$I_{b}$	Yes	Yes	Yes

## Data Availability

The data presented in this study are available on request from the corresponding author. The data are not publicly available due to institutional restrictions regarding experimental datasets collected at the University of Chile and the University of Santiago de Chile.

## Abbreviations

The following abbreviations are used in this manuscript:

SSCF	Stator Short-Circuit Faults
RIV	Residual Information Value
VSD	Variable Speed Drive
SFs	Stator Faults
FD	Fault Detection
ANNs	Artificial Neural Networks
RNNs	Recurrent Neural Networks
GRUs	Gated Recurrent Units
LSTM	Long Short-Term Memory
CNNs	Convolutional Neural Networks
DTC	Direct Torque Control
DFOC	Direct Field-Oriented Control
SVMs	Support Vector Machines
PSO	Particle Swarm Optimization
RPCNNet	Recurrence Plot Convolutional Neural Network
MPC	Model Predictive Control
DT	Decision Trees
MLP	Multilayer Perceptrons
FFT	Fast Fourier Transform
ANC-Net	Active Noise Cancellation Network
MRAS	Model Reference Adaptive System
CMV	Common-Mode Voltage
ANM	Additive Noise Model
MI	Mutual Information
EKF	Extended Kalman Filter
DSP	Digital Signal Processor
MCSA	Motor Current Signature Analysis
FAR	False Alarm Rate
SW	Sliding Window
EMI	Estimated Mutual Information
MSE	Mean Squared Error
PF	Power Factor
